# Genome-wide analysis of genetic diversity in *Anopheles darlingi* from Rondônia State, Brazil

**DOI:** 10.1038/s42003-025-09316-w

**Published:** 2025-12-04

**Authors:** Sophie Moss, Holly Acford-Palmer, Alice O. Andrade, Emilia Manko, Jody Phelan, Matthew Higgins, Jansen F. Medeiros, Taane G. Clark, Maisa S. Araujo, Susana Campino

**Affiliations:** 1 Faculty of Infectious and Tropical Diseases, London School of Hygiene and Tropical Medicine, London, UK; 2 Plataforma de Produção e Infecção de Vetores da Malária- PIVEM, Laboratório de Entomologia, Fiocruz Rondônia, Porto Velho, RO Brazil; 3 Instituto Nacional de Epidemiologia da Amazônia Ocidental – EpiAmO, Porto Velho, RO Brazil; 4 Faculty of Epidemiology and Population Health, London School of Hygiene and Tropical Medicine, London, UK; 5 Programa de Pós-Graduação em Conservação e uso de Recursos Naturais – PPGReN, Fundação Universidade Federal de Rondônia, Porto Velho, RO Brazil; 6 Laboratório de Pesquisa Translacional e Clínica, Centro de Pesquisa em Medicina Tropical, Porto Velho, RO Brazil

**Keywords:** Genetic markers, Evolutionary biology

## Abstract

*Anopheles darlingi* is the primary malaria vector in Central and South America and is responsible for most malaria transmission in the Amazon region. In this study, we perform whole-genome analysis of individual *An. darlingi* mosquitoes to explore genomic diversity, signatures of selection, and insecticide resistance markers. We analysed wild-caught (*n* = 20) and colony-maintained (*n* = 8) mosquitoes from the State of Rondônia, Brazil. In total, 1.54 million high-quality single-nucleotide polymorphisms (SNPs) were identified. Population genomic analysis revealed genetic differentiation between the colony and wild populations. No SNPs previously associated with insecticide resistance were detected. However, several SNPs were observed in four genes commonly associated with insecticide resistance: *ace1*, *rdl*, *gste2*, and *vgsc*. Genes under directional selection were identified, but no clear selective sweeps were found using genome-wide selection scans. Gene duplications were identified in cytochrome P450 genes, which are known to metabolise pyrethroids. This study provides a detailed genetic profile of *An. darlingi*, highlighting genes potentially involved in insecticide resistance, and presents an analysis of signatures of selection based on WGS data for this species. Our findings identify markers in insecticide resistance-associated genes that warrant further investigation through phenotypic-genotypic assays.

## Abstract (web summary)

Genome-wide analysis of the malaria vector *Anopheles darlingi* from Rondônia State, Brazil, explores genetic diversity, insecticide resistance, and signatures of natural selection in this mosquito species.

## Introduction

*Anopheles darlingi* (or *Nyssorhynchus darlingi*^1^) is a major malaria vector throughout South and Central America^2^. This species is an efficient vector of both *Plasmodium vivax* and *Plasmodium falciparum* malaria due to its high susceptibility to *Plasmodium* infection, its ability to maintain transmission at low parasite densities^2^, and its propensity to feed on humans^3,4^. Furthermore, *An. darlingi* is highly adaptable and exhibits significant behavioural variation, including the ability to adapt to environments undergoing urbanisation and deforestation^2,5^. This species is particularly prominent in recently deforested areas, where its abundance surpasses that of other *Anopheles* species^6^. The ongoing anthropisation of these environments is thought to sustain malaria transmission by increasing the availability of larval habitats for *Anopheles* vectors^7,8^.

In 2023, an estimated 500,000 malaria cases were reported in the Americas, with Brazil accounting for the largest share (~163,000 cases), predominantly in the Amazon region^9^. Malaria cases in Brazil have increased from 138,000 in 2015, with *P. vivax* responsible for 72.1% of infections and *P. falciparum* for 16%^10^. Alarmingly, the proportion of *P. falciparum* cases has risen by 7%^11^, raising concerns due to its higher mortality rate, particularly among children and pregnant women. Strengthening malaria control in Brazil is crucial to curbing transmission and achieving both the country’s National Elimination Plan and the World Health Organization’s (WHO’s) goal of a 90% reduction in malaria incidence by 2030^12^.

Malaria control in Brazil primarily relies on indoor residual spraying (IRS) and insecticide-treated nets (ITNs)^13,14^. Pyrethroids are the most widely used insecticides against *Anopheles* mosquitoes, while carbamates and organophosphates are deployed for *Aedes* control in arbovirus programmes^15,16^. Systematic insecticide resistance surveillance is essential for informed vector control strategies, yet Brazil currently lacks a national screening programme for *Anopheles* mosquitoes. Resistance to pyrethroids, carbamates, and organochlorines has been documented in *An. darlingi* populations in Bolivia, Colombia, French Guiana, and Peru^17,18^. Insecticide resistance has not been reported in Brazil, which is likely due to limited historical surveillance^17^.

Insecticide resistance arises through multiple mechanisms, including target-site mutations, metabolic resistance, behavioural adaptations, cuticle thickening, and microbiome alterations^19,20^. Four key genes associated with insecticide resistance are the voltage-gated sodium channel gene (*vgsc)*, the acetylcholinesterase gene (*ace1)*, the glutathione S-transferase gene (*gste2)*, and the gamma-aminobutyric acid (GABA) receptor (*rdl –* resistant to dieldrin gene). The *vgsc* gene encodes a transmembrane protein essential for nerve function, and is targeted by pyrethoid and dichlorodiphenyltrichloroethane (DDT) insecticides^21^. Whereas, organophosphate and carbamate insecticides work by inhibiting acetylcholinesterase (encoded by *ace1*), which is required for stopping synaptic transmission by hydrolyzing the acetylcholine neurotransmitter^22^. The *gste2* gene encodes a metabolic enzyme able to metabolise insecticides^23^, and the *rdl* gene encodes a GABA subunit which is involved in nervous system signalling^24^. Target-site resistance is often driven by single-nucleotide polymorphisms (SNPs) in these genes. Well-characterised resistance-associated SNPs in the *Anopheles gambiae* sensu lato (s.l.) complex include V402L, L995F/S, I1527T, F1529C and N1570Y in the voltage-gated sodium channel (*vgsc*) linked to pyrethroid and DDT resistance, G280S in *ace1* conferring organophosphate and carbamate resistance^25–27^, L119F in *gste2* associated with DDT/pyrethroid resistance^23^, and A296G/S, V327I and T345S in the gamma-aminobutyric acid (GABA) receptor (*rdl*), which have been associated with resistance to dieldrin^28,29^. These SNPs may be identified rapidly using multiplex amplicon sequencing^30,31^. However, no SNPs associated with target-site resistance in other *Anopheles* species have been identified in *An. darlingi* to date^32–36^. Metabolic resistance, which results from differential gene expression enhancing detoxification enzyme activity^23,37–39^, is hypothesised to play a dominant role in insecticide resistance in *An. darlingi*, particularly given the absence of known target-site mutations. Increased metabolic resistance can arise from increased copy number of metabolic enzymes, resulting in increased gene expression. Three key metabolic enzyme families include the cytochrome P450s, esterases, and glutathione-S-transferases (GSTs)^40^. Copy number variation (CNVs) in cytochrome P450 genes is highly complex^41,42^, and pyrethroid resistance in *Anopheles* mosquitoes has been strongly associated with CNVs in *cyp6aa1*^43^*, cyp6aap*^44^*, cyp6m2, cyp6p3, cyp6z1* and *cyp9k1*^45,46^, and with CNVs in the GST encoding gene *gste2*^47^. Furthermore, significant overexpression of genes in these metabolic families has been associated with insecticide resistance^48–50^_._

Previous molecular studies of *An. darlingi* have focused on screening a limited number of loci in four candidate resistance genes (*vgsc, ace1, gste2, rdl*). However, genome-wide approaches are needed to identify novel resistance markers^30,36^. Whole-genome sequencing (WGS) has been extensively applied to *An. gambiae* s.l. mosquitoes to investigate population structure and resistance evolution^51,52^. Here, we apply WGS to two Brazilian *An. darlingi* populations in Rondônia State: a wild population from Candeias do Jamari, and a colony population from Porto Velho, both in the State of Rondônia and < 100 km apart from each other. By examining ancestry, population structure, and genetic regions under selection, we aimed to characterise genomic diversity and identify molecular markers potentially associated with insecticide resistance in this vector population, serving as a valuable resource for future research on vector adaptation and resistance mechanisms.

## Results

### Whole genome sequence data for *An. darlingi*

Twenty-eight *An. darlingi* mosquito samples were sequenced using WGS, yielding an average genome-wide coverage of 8.99-fold. The dataset comprised 20 wild-caught specimens from Candeias do Jamari (Rondônia) and 8 colony samples derived from mosquitoes originally maintained in Porto Velho (Rondônia) in 2018. After variant calling and quality filtering, 1,539,646 high-quality variants were retained for analysis, with 1,489,593 variants detected in the wild-caught samples and 535,667 in the colony samples. This discrepancy in variant number is likely due to the difference in sample size between these two populations.

### *An. darlingi* colony samples are genetically distinct from wild populations

**Fig. 1:**
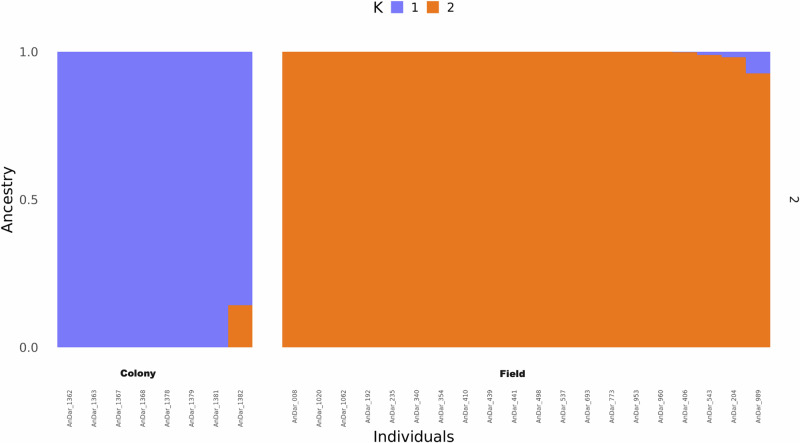
Ancestry analysis of *An. darlingi* colony (*n* = 8) and wild (*n* = 20) mosquitoes. The number of ancestral populations was estimated to be 2, and these are denoted as K1 and K2.

Admixture analysis identified two ancestral groups (K = 2), designated as K1 and K2, which differentiate the colony (n = 8) and wild (n = 20) Rondônia populations (Fig. 1). One colony sample (AnDar_1382) exhibited mixed ancestry from both K1 and K2, while three wild-caught samples primarily belonged to K2 but contained a small proportion of K1 ancestry.

Principal component analysis (PCA) reveals a clear genetic distinction between the colony and wild mosquitoes, consistent with the admixture analysis (Supplementary Fig. 1). As expected, chromosome-specific PCA highlights the separation between colony and wild mosquitoes across chromosomes 2, 3, and X (Supplementary Fig. 1). The mitochondrial PCA, and a maximum likelihood tree based on the mitochondrial genome, did not show clear distinction between the colony and wild mosquitoes (Supplementary Figs. 1 and 2).

Population differentiation due to genetic structure was measured using the Fixation index (F_ST_) statistic, where a value of 1 indicates complete differentiation between two populations, whereas a value of 0 indicates no differentiation^53^. F_ST_ analysis calculated in 1000 bp windows revealed allele frequency differences between the two populations. On chromosome 2, 349 SNPs had an F_ST_ ≥ 0.75, including 25 SNPs with an F_ST_ ≥ 0.9 and 3 SNPs with an F_ST_ of 1. On chromosome 3, 63 SNPs had an F_ST_ ≥ 0.75, with 8 of these reaching F_ST_ ≥ 0.9. Chromosome X had 3 SNPs with an F_ST_ ≥ 0.75, while no SNPs on the mitochondrial genome had F_ST_ ≥ 0.75 (F_ST_ on the mitochondrial genome ranged from 0.062 to 0.419). Genomic windows and genes with F_ST_ ≥ 0.9 across the genome are summarised in Table 1. Median genome-wide F_ST_ between the wild-caught and colony Rondônia *An. darlingi* populations was 0.143. This population differentiation is markedly high in comparison to other *Anopheles* species; low divergence is common in *An. gambiae* and *An. coluzzii* (F_ST_ < 0.05)^54^, and even F_ST_ computed between these two species has been identified as low (F_ST_ < 0.14)^51^. Similarly high *An. darlingi* F_ST_ values were identified in a larger study of 1,094 mosquitoes, where the median pairwise F_ST_ between populations in Brazil, Colombia, Guiana, Guyana, Peru, and Venezuela was 0.18^55^. These high *An. darlingi* F_ST_ values indicate increased genetic separation between populations, in comparison with African *Anopheles* species.

**Table 1:** Genes within genomic windows (1000 bp) with F_ST_ scores ≥ 0.9

Chr.	Genomic Window Start Position	Genomic Window End Position	F_ST_ Score	Gene name (encoded protein)
2	18740129	18741128	0.930	LOC125948827 (myb-like protein AA); ENSADAG00000001074 (ZNF281)
2	18746129	18747128	0.927	LOC125948827 (myb-like protein AA); ENSADAG00000001074 (ZNF281)
2	19057129	19058128	0.901	LOC125948822
2	19774129	19775128	0.954	LOC125948886 (neuropeptide CCHamide-2 receptor-like)
2	21131129	21132128	0.911	LOC125948306 (hemicentin-1); ENSADAG00000000808
2	21176129	21177128	0.924	LOC125948306 (hemicentin-1); ENSADAG00000000808
2	21397129	21398128	1	LOC125959064 (trithorax group protein osa)
2	21655129	21656128	1	LOC125959087 (allatostatin-A receptor-like); ENSADAG00000005016
2	22053129	22054128	0.931	LOC125948806; ENSADAG00000003076 (MIDEAS)
2	23321129	23322128	0.913	LOC125950219
2	23940129	23941128	0.934	LOC125951741 (mitochondrial import inner membrane translocase subunit TIM14); ENSADAG00000002236 (DNAJC19)
2	24109129	24110128	0.976	LOC125952255 (uncharacterised)
2	24381129	24382128	0.914	LOC125952268 (neuroligin-4, Y-linked)
2	24659129	24660128	0.923	LOC125951300 (gamma-aminobutyric acid type B receptor subunit 2); ENSADAG00000000746
2	25002129	25003128	0.933	LOC125947887 (5’-3’ exoribonuclease 1); ENSADAG00000002912 (XRN1)
2	75896129	75897128	0.904	LOC125949681 (histone demethylase UTY); ENSADAG00000003908 (MAML1)
2	81022129	81023128	0.923	LOC125949814; ENSADAG00000002381
3	4853596	4854595	0.933	LOC125956255 (teneurin-m)
3	9653596	9654595	0.913	LOC125954234 (neuroglobin-like)
3	69666596	69667595	0.923	LOC125956843 (uncharacterised)
3	70008596	70009595	0.904	LOC125956845 (diacylglycerol kinase 1)
3	70347596	70348595	0.909	LOC125955750 (rho GTPase-activating protein gacF); ENSADAG00000009704 (plppr4b)

*An. darlingi* colony (*n* = 8) and wild (*n* = 20) mosquitoes.

### Mutations in genes associated with insecticide resistance

SNPs were identified in four key genes associated with insecticide resistance in other vector species (*ace-1, gaba, GSTe2*, and *vgsc*) (Supplementary Table 1). Seven nonsynonymous SNPs were detected in these four genes (Table 2). However, none of the resistance mutations that have previously been associated with insecticide resistance in *Anopheles* species were detected. An additional 129 potential genes of interest were investigated, including those where reported SNPs have been associated with reduced insecticide efficacy. These included *vgsc, ace-1*, all *gaba* genes, cytochrome P450s, carboxylesterases, glutathione transferases, and glucuronyltransferases. In total, 442 nonsynonymous SNPs were identified across these genes (Supplementary Data 1**)**. The T283K mutation in the ADAR2_008159 gene (*cyp6aa1* in *An. funestus*) has been identified as possibly linked to deltamethrin resistance in *An. darlingi*^55^. This mutation was not identified in the Rondônia samples. However, two other non-synonymous mutations were identified in this gene: I370V at 43.8% allelic frequency in the wild population and 31.3% in the colony population, and E359Q, which was found at 8.8% allelic frequency in the wild population and was absent in the colony population.

**Table 2:** Non-synonymous mutations identified in the four key insecticide resistance genes

Gene	Chr	Position	Ref	Alt	Codon Change in *An. darlingi*	Codon in *An. gambiae* PEST	Codon in *Musca domestica*	Alternate Allele Frequency %	
								Wild-caught (*n* = 20)	Colony (*n* = 8)
*ace1*	2	15676267	T	C	Val32Ala^a^	Ala27^b^	-	56.7	50.0
*gste2*	2	89825712	A	G	Asn82Ser^c^	Asp82^d^	Asp82^i^	2.94	0
*gste2*	2	89825747	G	A	Ala94Thr^c^	Ala94^d^	Ala94^i^	13.3	0
*gste2*	2	89825962	G	A	Glu142Lys^c^	Glu142^d^	Glu142^i^	2.94	0
*gste2*	2	89827309	G	C	Val276Leu^c^	Val57^d^	Thr57^i^	83.3	50.0
*vgsc*	3	35318429	C	T	Pro523Leu^e^	Pro509^f^	Pro529 ^j^	2.94	0
*rdl*	3	53427108	G	A	Ser90Asn^g^	Ser90^h^	Ser93^k^	3.33	0

*An. darlingi* colony (*n* = 8) and wild (*n* = 20) mosquitoes.

^a^Codon numbering according to *Anopheles darlingi* transcript XM_049679441.

^b^Codon numbering according to *Anopheles gambiae* transcript XM_061649305.

^c^Codon numbering according to *Anopheles darlingi* transcript XM_049692446.

^d^Codon numbering according to *Anopheles gambiae*transcript AGAP009194-PA.

^e^Codon numbering according to *Anopheles darlingi* transcript XM_049687134.

^f^Codon numbering according to *Anopheles gambiae* transcript AGAP004707-PB.

^g^Codon numbering according to *Anopheles darlingi* transcript XM_049684483.

^h^Codon numbering according to *Anopheles gambiae* transcript AGAP006028-PB.

^I^Codon numbering according to *Musca domestica* transcript XP_058986999.

^j^Codon numbering according to *Musca domestica* transcript ARX79626.

^k^Codon numbering according to *Musca domestica* transcript NP_001292048.1.

### Nucleotide diversity

Nucleotide diversity (π) is the average number of nucleotide differences per site, between two populations^56^. π was calculated to estimate the level of genetic variation within the wild mosquito population, and was computed in 100 bp windows across the genome. The average nucleotide diversity among these 20 *An. darlingi* was π = 0.002, which is lower than has previously been found in *An. gambiae s.l*. populations from 15 locations across Africa (averaging at π = 0.015)^51^, and likely reflects this small sample size. Nucleotide diversity in key insecticide resistance genes was compared between the wild-caught *An. darlingi* (*n* = 20) and a reference dataset of wild-caught *An. gambiae* sensu stricto (s.s.) samples (*n* = 42) from Bubaque Island in the Bijagós Archipelago, Guinea-Bissau, West Africa (Table 3)^57^. This reference dataset was selected for comparison as both are wild *Anopheles* populations caught in a small geographic area. Nucleotide diversity was low in both populations but consistently 3 to 6 times higher in the wild-caught *An. gambiae* s.s. from Bubaque Island than the wild-caught *An. darlingi* (Table 3), indicating relatively little variation within this small sample of wild-caught *An. darlingi* from Rondônia State.

**Table 3:** Nucleotide diversity (π) calculated in 100 bp windows across key insecticide resistance genes for wild-caught Rondônia State mosquitoes (*n* = 20) and wild-caught Bijagós Archipelago mosquitoes (*n* = 42)

Gene	Wild-caught *An. darlingi* (*n* = 20) (Rondônia State)	Wild-caught *An. gambiae* sensu stricto (*n* = 42) (Bijagós Archipelago*)
*ace1*	0.004	0.012
*gste2*	0.003	0.011
*rdl*	0.004	0.017
*vgsc*	0.001	0.006

*Bubaque Island.

### Natural selection across the *An. darlingi* genome

**Fig. 2:**
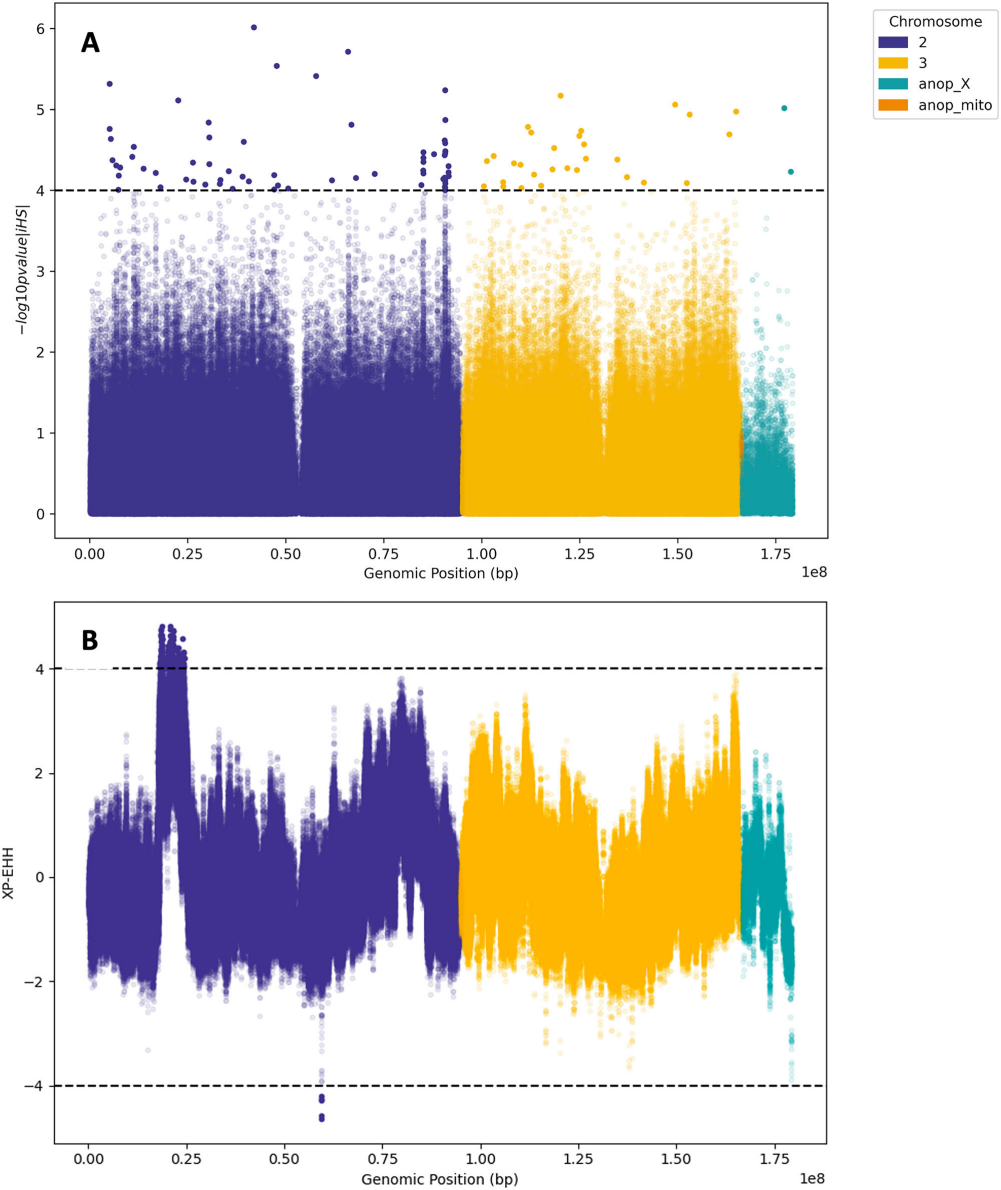
Directional Selection Analysis. **A** iHS scores for wild-caught mosquito samples (*n* = 20) where iHS score ≥ 4 indicates positive selection. This significance threshold is depicted by the black dashed line. **B** XP-EHH comparison between colony mosquitoes (*n* = 8) and wild (*n* = 20) mosquitoes. Values ≤ -4 indicate significant selection in the wild-caught samples when compared to the colony mosquitoes, whereas values ≥ 4 indicate significant selection in the colony mosquitoes when compared to the wild samples.

Integrated haplotype (iHS) and Garud’s H_12_ statistics were used to identify loci under directional selection in the wild samples (*n* = 20). The iHS metric measures extended haplotype homozygosity to identify signatures of recent selection within a particular population^58^. A total of 103 loci showed evidence of directional selection, which may be due to selection pressure from insecticide use, or from other selection pressures (iHS score ≥ 4) **(**Fig. 2A**)**. Of the 103 loci with significant iHS scores, 57 were in protein-coding genes (Table 4). None of these protein-coding genes have previously been associated with insecticide resistance in *An. darlingi* or other *Anopheles* species.

**Table 4:** Protein coding genes under significant selection in the wild-caught (*n* = 20) mosquito population (iHS score ≥ 4)

Chr.	Gene name	Description	iHS score
X	LOC125948124	Wilms tumour protein 1-interacting protein-like	5.02
2	LOC125950173	Mucin-5AC-like	5.11
2	LOC125949705	RNA polymerase-associated protein CTR9 homologue	4.65
2	LOC125948229	Elongation factor-like GTPase 1	4.64
2	LOC125952105	Uncharacterized protein-coding gene	4.58
2	LOC125950338	Furin-like protease 2	4.54
2	LOC125950341	Uncharacterised protein-coding gene	4.41
2	LOC125951977	Axin	4.37
2	LOC125959173	GS homeobox 1-like	4.33
2	LOC125951112	Conserved oligomeric Golgi complex subunit 3	4.28
2	LOC125950864	Hemicentin-1	4.27
2	LOC125950282	Autophagy-related protein 16-1	4.24
2	LOC125951565	Ras-related and oestrogen-regulated growth inhibitor-like protein	4.24
2	LOC125952875	Klarsicht protein	4.23
2	LOC125948116	Uncharacterised protein CG43867	4.22
2	LOC125950707	RNA-binding protein 34-like	4.19
2	LOC125951134	Uncharacterized protein CG45076-like	4.18
2	LOC125952079	Protein spaetzle 3	4.16
2	LOC125952297	Dual specificity calcium/calmodulin-dependent 3'2C5’-cyclic nucleotide phosphodiesterase 1A-like	4.15
2	LOC125952077	Protein star	4.15
2	LOC125952109	Uncharacterised protein-coding gene	4.14
2	LOC125951278	EH domain-containing protein 1	4.13
2	LOC125947933	Nuclear pore complex protein DDB_G0274915-like	4.11
2	LOC125949291	Segment polarity protein dishevelled	4.11
2	LOC125952098	Estradiol 17-beta-dehydrogenase 11	4.09
2	LOC125950428	Tyrosine-protein kinase Dnt-like	4.07
2	LOC125952064	CD109 antigen-like	4.04
3	LOC125954982	Glucose transporter type 1	5.06
3	LOC125956844	Netrin receptor DCC	4.97
3	LOC125957598	Uncharacterised protein-coding gene	4.94
3	LOC125953375	Pericentrin	4.79
3	LOC125956438	Neuronal growth regulator 1-like	4.74
3	LOC125953484	Zwei Ig domain protein zig-8-like	4.71
3	LOC125954775	Protein bric-a-brac 1-like	4.7
3	LOC125956437	Succinate dehydrogenase [ubiquinone] flavoprotein subunit 2 C mitochondrial-like	4.57
3	LOC125957719	Zwei Ig domain protein zig-8	4.38
3	LOC125958263	Transmembrane channel-like protein 7	4.33
3	LOC125954270	Protein TIS11	4.28
3	LOC125953928	Sodium-dependent transporter bedraggled	4.24
3	LOC125953389	Receptor-type guanylate cyclase Gyc76C-like	4.19
3	LOC125957210	Semaphorin-2A-like	4.16
3	LOC125954152	Hexokinase type 2	4.1
3	LOC125954661	Ubiquitin-conjugating enzyme E2 W	4.1
3	LOC125957447	Calcium-binding protein E63-1	4.09
3	LOC125954157	Mucin-5AC-like	4.05

Garud’s H_12_ statistic examines the frequency of the most common haplotype in a population (H_1_) versus the second most common haplotype (H_2_), to identify soft and hard selective sweeps^59^. H_12_ was also calculated as an additional method to identify selective sweeps across the genome^60^. No significant sweeps were identified (Supplementary Fig. 3).

Cross-population extended haplotype homozygosity (XP-EHH) compares selection between two populations by contrasting haplotype lengths associated with the same allele in different populations^61^. XP-EHH analysis was performed to identify loci under significant positive directional selection in the wild mosquitoes when compared to colony mosquitoes **(**Fig. 2B**)**. A value ≤ -4 indicates significant selection in the wild-caught samples when compared to the colony mosquitoes, whereas a value ≥ 4 indicates significant selection in the colony mosquitoes. Protein-coding genes identified to be under significant selection are listed in Table 5. Notably, loci under significant selection in wild mosquitoes, compared to colony mosquitoes, were located outside of protein-coding genes.

**Table 5:** Protein-coding genes under significant selection in the colony mosquitoes (*n* = 8) when compared to the wild-caught (*n* = 20) Rondônia State mosquitoes

Gene	Description	XP-EHH value
LOC125948167	forkhead box protein O	4.06
LOC125950636	myosin-10	4.12
LOC125948887	flotillin-2	4.75
LOC125948913	microtubule-associated protein Jupiter	4.38
LOC125948827	myb-like protein AA	4.81
LOC125948822	uncharacterized LOC125948822	4.06
LOC125948826	nephrin	4.22
LOC125948920	3’-5’ ssDNA/RNA exonuclease TatD	4.15
LOC125948424	protein crumbs	4.22
LOC125948318	hybrid signal transduction histidine kinase M	4.22
LOC125952005	uncharacterized LOC125952005	4.82
LOC125948312	apolipoprotein D-like	4.04
LOC125948301	inactive rhomboid protein 1-like	4.08
LOC125948302	RNA exonuclease 1 homologue	4.34
LOC125948306	hemicentin-1	4.58
LOC125959064	trithorax group protein osa	4.35
LOC125959072	fibroblast growth factor receptor homologue 1-like	4.42
LOC125950577	octopamine receptor beta-3R-like	4.73
LOC125950168	uncharacterized LOC125950168	4.40
LOC125950219	uncharacterized LOC125950219	4.16
LOC125950181	protein groucho	4.05
LOC125950219	uncharacterized LOC125952255	4.59
LOC125948441	heart- and neural crest derivatives-expressed protein 2-like	4.05
LOC125948435	uncharacterized	4.32

All genes identified under significant selection were in chromosome 2.

To further investigate signatures of selection, Tajima’s D (T_D_) statistic was calculated in 20,000 bp windows across the genome for each population. In the colony samples, median T_D_ was 1.09, and 269 windows exhibited T_D_ values > 2.5 (Supplementary Fig. 4), suggesting balancing selection or a recent population bottleneck; consistent with the characteristics of a colony-bred population. Whereas the wild-caught samples had a median T_D_ of -0.877, suggesting possible population expansion. This is lower than the T_D_ found in a larger study of 1094 *An. darlingi* from across South America (median T_D_ = 0.26 per population), suggesting stability in *An. darlingi* population size^55^. Whereas, our lower T_D_ value supports a possible population expansion in the wild-caught mosquitoes from Rondônia, which may be influenced by our small sample of 20 wild-caught mosquitoes. Both of these studies identified T_D_ values contrasting with the more negative T_D_ associated with *An. gambiae* populations, which are reported as T_D_ < -1.5 and reflect rapid population expansion within this African species complex^51^.

### Structural variants

Structural variants (SVs) were identified in regions containing putative genes of interest, including 148 inversions, 54 deletions, 27 duplications, 18 insertions, and 32 inter-chromosomal translocations (Supplementary Data 2). Among these SVs, we identified gene duplications which frequently involved members of the cytochrome P450 gene family (Table 6); a gene family previously associated with insecticide resistance^41,48^. These included cytochrome P450 *4cs3-like*, *4d1*-*like*, *4C1*-like and *3A19*-*like* genes, and cytochrome P450 *6a13*, *313a4*, and *313a1* genes. Additionally, we identified duplications that introduced premature stop codons, splice region variants, and duplicated exons. One limitation of this analysis is the relatively low average read depth (8.99-fold), which may have resulted in missed SVs that could be detected in future studies using higher coverage sequencing.

**Table 6:** Duplications with high impact on genes that may be involved in insecticide resistance

Chr	Duplication ID	Genes with HIGH impact as defined using SnpEff^90^ and the effect on these genes.	Frequency in wild-caught Rondônia State mosquitoes (%)	Frequency in colony mosquitoes (%)
X	DUP00004667	Frameshift and splice region variant in the cytochrome P450 4c3-like gene (LOC125953818)	14.7	0
2	DUP00010794	Gene fusion involving two cytochrome p450 4d2-like genes (LOC125950881 and LOC125950889. Duplication between 14392190 and 14395703 (~3.5 kb).	26.3	12.5
2	DUP00010862	Introduction of a premature stop codon and impacting a splice region in cytochrome P450 4d2-like gene (LOC1259483340)	36.1	12.5
2	DUP00019559	Gene fusion between the cytochrome P450 4C1-like gene (LOC125950286) and the transmembrane and coiled-coil domain 2 protein (LOC125950273). Duplication between positions 36277503 and 36280083 (~2.5 kb).	0	31.3
2	DUP00019560	Gene fusion between transmembrane and coiled-coil domain 2 protein (LOC125950273) and the cytochrome P450 4C1-like gene (LOC125950286). Gene fusion between LOC125950273 and the cytochrome P450 4C1-like gene (LOC125950311). Duplication between positions 36278078 and 36281104dup, ~3 kb.	52.5	50.0
2	DUP00019562	Gene fusion between transmembrane and coiled-coil domain 2 protein (LOC125950273) and the cytochrome P450 4C1-like gene (LOC125950286). Gene fusion between LOC125950273 and the cytochrome P450 4C1-like gene (LOC125950311). Duplication between positions 36278947 and 36282009dup, ~3.1 kb).	31.6	18.8
2	DUP00044715	Gene fusion between multidrug resistance-associated protein 1-like gene (LOC125951334) and ras-related protein Rab-9B (LOC125951567). Duplication of part of the glutathione S-transferase-1 like gene (LOC125951365).	2.5	0
2	DUP00048534	Gene fusion between cytochrome P450 9f2s (LOC125952882 and LOC125952880). Duplication between positions 91682639 and 91685220, ~2.5 kb.	2.78	43.75
3	DUP00052410	Gene fusion between two probable cytochrome P450 6a13 genes, LOC125956304 and LOC125958131. Duplication between positions 5372527 and 5375351 (~2.8 kb).	17.65	6.25
3	DUP00072025	Gene fusion between probable cytochrome P450 313a4 (LOC125955537) and probable cytochrome P450 313a1 (LOC125955540) Duplication between positions 46108971 and 46118421 (~9.4 kb).	41.7	43.8
3	DUP00072031	Duplication of cytochrome P450 3A19-like (LOC125955539), and a frameshift variant of this gene. Gene fusion between cytochrome P450 3A19-like (LOC125955539), probable cytochrome P450 313a4 (LOC125955538), probable cytochrome P450 313a4 (LOC125955537), and probable cytochrome P450 313a1 (LOC125955540)	13.9	25.0
3	DUP00075794	Gene fusion between glutamate receptor 1-like (LOC125954300) and an uncharacterized protein (LOC125954378). Duplication between positions 52862921 and 53509817, ~647 kb.	2.5	0

*An. darlingi* colony (*n* = 8) and wild (*n* = 20) mosquitoes.

## Discussion

This study presents WGS data and analysis for two *An. darlingi* populations from Rondônia, Brazil: a wild-caught population from Candeias do Jamari and a colony population originated from mosquitoes collected in Porto Velho. Clear genetic separation between these two populations was identified in the principal component and admixture analyses, with distinct ancestries and differentiation across chromosomes 2, 3, and X. Maximum likelihood phylogenetic analysis and principal component analyses of mitochondrial DNA did not show clear separation between the colony and wild-caught populations, which is not surprising given the close proximity of the Candeias do Jamari and Porto Velho collection sites (<100 km apart). Nucleotide diversity was low in both populations, reflecting the small sample size, and Tajima’s D analysis suggested a bottleneck in the colony mosquitoes and possible population expansion in the wild-caught mosquitoes. These findings align with expectations for the colony, given extensive inbreeding, and for the wild-caught population, which was sampled from a small geographic area (radius <5 km).

Fixation index (F_ST_) analysis further supported genetic differentiation between the two populations, with chromosome 2 showing the highest divergence, followed by chromosome 3 and chromosome X. Whereas, F_ST_ analysis of the mitochondrial genome showed reduced divergence. Notably, the gamma-aminobutyric acid type B receptor subunit 2 (ADAR2_002857) on chromosome 2 exhibited high differentiation between the colony and wild-caught samples. Organochlorine insecticides act by binding to the GABA receptor complex, with the most common resistance mutation being A259S/G in the *rdl* gene^29,62^. Overall, F_ST_ was high in *An. darlingi* when compared with other *Anopheles* species, including *An. gambiae* and *An. coluzzii* in Africa^51^. This high F_ST_ is corroborated by a much larger study of 1094 *An. darlingi* from across South America, which also found elevated F_ST_ scores in *An. darlingi* population comparisons^55^.

Previous studies using mitochondrial *cox-1* sequences have suggested that geographic barriers, such as the Andes and Amazon River, influence *An. darlingi* population structure^63–66^. The colony samples in this study originated from mosquitoes collected in Porto Velho in 2018, while the wild-caught samples were obtained less than 100 km away in 2018 and 2019^67^. Our study demonstrates the advantage of genome-wide approaches in resolving population dynamics as, despite their geographic proximity, WGS analysis was able to effectively distinguish the ancestries of these populations.

No well-characterised mutations associated with insecticide resistance in other *Anopheles* species were detected in *An. darlingi*. However, we identified seven novel nonsynonymous SNPs within four key resistance-associated genes (*ace-1, rdl, GSTe2, vgsc*), including the Pro523Leu mutation in *vgsc*. Further research is needed to determine their functional significance, including the integration of phenotypic resistance data. Similar investigations in *An. funestus* have identified molecular markers of resistance, suggesting a comparable approach could be applied to *An. darlingi*^38,68,69^.

Metabolic detoxification is hypothesised to play a greater role in *An. darlingi* insecticide resistance than target-site mutations^70^. In *An. gambiae*, pyrethroid resistance has been linked to copy number variations (CNVs) in detoxification enzyme families (cytochrome P450s, esterases, and glutathione-S-transferases (GSTs)). Using WGS, we were able to examine the genetic diversity of these gene families in *An. darlingi*. We found no selective sweeps over insecticide resistance-related genes through analysis of extended haplotype homozygosity using H_12_^60^. However, structural variant analysis revealed high-impact gene duplications involving multiple cytochrome P450 genes:*cyp4c3, cyp4d2, cyp4c1, cyp9f2s, cyp6a13, cyp313a4, cyp3a19, cyp313a1-like*. Three of these genes (*cyp4c1, cyp4c3, cyp4d2*) have been implicated in insecticide resistance in other species^71,72^; *cyp4c1* is upregulated in *Aedes aegypti* resistant to DDT, pyrethroids, and carbamates, and in *Cydia pomonella* larvae resistant to deltamethrin^73,74^. Additionally, *cyp4c1* has been linked to metabolic compensation during starvation in cockroaches and is hormonally regulated^75,76^. It has also been found to be upregulated in the crop pest *Leptinotarsa decemlineata* following exposure to neonicotinoids^77^. In contrast, the function of *cyp4c3* remains less well understood, though it has been found upregulated in honeybees after amitraz insecticide treatment^78^.

In addition, we identified a duplication in the glutathione S-transferase gene (ADAR2_012021), a gene family known to confer resistance to organophosphates and DDT^79^. Class 1 GSTs, which mediate resistance in *Musca domestica*, are believed to perform a similar function in *Anopheles* species, though further evidence is needed to confirm this^80–82^. More generally, to determine the functional role of these duplications in insecticide resistance, further studies incorporating phenotypic resistance data are required. Comparative RNA-seq analysis of insecticide-resistant and susceptible *An. darlingi* populations could help to identify resistance-associated gene expression changes. Limitations of this study include the lack of phenotypic resistance data and the small overall sample size, which is limited to a small geographic region. Future studies should incorporate larger sample sizes of *An. darlingi* from diverse regions, along with phenotypic resistance data for the most frequently used insecticides in Brazil. Other phenotypes can also be investigated, such as biting behaviour, which has been explored for this species using low-coverage genomic sequencing data of pooled samples^83^.

Overall, our study presents an initial analysis of WGS data from *An. darlingi* malaria vectors, establishing a foundation for future studies with more expansive sample sizes and associated phenotypic data. Large-scale surveillance of *An. darlingi* is essential to gain deeper insights into its molecular ecology and to identify genomic markers linked to key traits, including insecticide resistance, vector competence, blood-feeding behaviour, and adaptation to environmental changes. This comprehensive approach will provide valuable insights into the genetic factors influencing *An. darlingi* populations and enhance the development of targeted control strategies.

## Methods

### Mosquito collection, species identification, and DNA extraction

Two populations of *An. darlingi* from the State of Rondônia were used in this study, one wild-caught population from Candeias do Jamari, right margin of Madeira River, (*n* = 20), and a cohort of colony isolates from Porto Velho (*n* = 8)^67^. The wild-caught mosquitoes were collected during vector density studies in malaria-endemic regions in Rondônia in 2018–19. Entomological surveys were performed as 8 consecutive collections at the beginning of the rainy season (Oct and Nov 2018), and another eight collections were performed at the beginning of the dry season (May and June 2019)^84^. The colony of mosquitoes has been maintained since 2018, originally collected in Porto Velho, left margin of Madeira River^45^. Specimens were morphologically identified via microscopy. Genomic DNA from the isolates was extracted from the whole mosquito using Qiagen DNaeasy Blood and Tissue kits (Qiagen, Hilden, Germany), according to the manufacturer’s instructions. DNA was quantified using the Qubit 2.0 fluorimeter HS DNA kit (Thermofisher), and stored at −20 °C.

### Whole genome sequencing and bioinformatics analysis

Twenty-eight *An. darlingi* isolates were sequenced using the Illumina MiSeq on 2 x 250 bp paired-end configuration in 2024, with an average read-depth of 8.99-fold. The raw paired fastq files were trimmed using *trimmomatic* software (version 0.39), and then aligned using bwa-mem software to the AnoDar_H01 (*An. darlingi)* reference genome using default parameters^85,86^. Genome coverage from the mapped bam files was calculated using *samtools*, and variants called and validated using *HaplotypeCaller* and *VailidateVariants* from GATK software, respectively (v4.1.4.1)^87,88^. Once VCFs had been generated for each sample, GATK was used to create a multi-sample VCF with *GenomicsDBImport* and *GenotypeGVCF* functions. The multi-sample VCF was filtered to include chromosomal variants using *bcftools* (v 1.17) and GATK’s *VariantFiltration*, with parameters: QD > 5.0, QUAL > 30.0, SOR < 3.0, FS < 60.0, MQ > 40.0, MQRankSum > − 12.5, ReadPosRankSum > -8.0. Reads were subsequently filtered to retain those with DP > 5.0 and GQ > 20.0, and variants were filtered to retain those with < 20.0% missing genotypes or MAF > 0.01. The resulting VCF was phased with *Beagle* (version 5.2)^89^ and annotated using *SnpEff* (v5.1 d)^90^.

### Population genetic analysis

From the filtered multi-sample VCF, a pairwise-genetic distance matrix was generated using PLINK (v1.90b6.21 64-bit)^91,92^. This matrix was used for the generation of a maximum-likelihood tree using RAxML-NG (v 1.2.0), and principal component analysis was calculated using the R package *ape* (5.7-1)^93^. The ML tree generated was annotated and visualised in iTOL^94^. Ancestry admixture analysis was conducted using ADMIXTURE software (version 1.3)^95^. The optimum K value (estimated number of ancestral populations) was calculated through cross-validation of 1-10 eigenvalue decay dimensions^92^. In this instance, K = 2 was estimated, and ADMIXTURE software was used to analyse the shared ancestral populations (denoted K1 and K2) in these samples. The output was then visualised in R using *ggplot2* package (v 3.4.2)^96^.

Genomic regions under selection were identified with the python package *scikit*-*allel* (v 1.3.6)^97^. Three complementary statistics were used: H_12_^60^, iHS, and XP-EHH^61,98^. The Integrated Haplotype Statistic (iHS) was used to find directional selection within populations, and extended haplotype homozygosity (XP-EHH) between the two populations^99,100^. Garud’s H12 was calculated using phased biallelic SNPs in 1000 bp windows, using the *moving*_*garud*_*h* function^97^. Two hundred iterations of H12 were calculated, followed by plotting of the mean value for each genomic window. iHS was computed using phased biallelic SNPs using the *allel.ihs* function^97^. Raw iHS scores were then standardized using the function *allel.standardize_by_allele_count*, and *p*-values were plotted. XP-EHH was calculated using phased biallelic SNPs using the *allel.xpehh* function. The Tajima’s D (T_D_) statistic was calculated using *vcftools* (v0.1.16) in 20 kbp windows across chromosomes to identify balancing selection^101,102^. Nucleotide diversity (π) was calculated in 100 bp windows across chromosomes using *vcftools* and plotted in R. Weir and Cockerham’s F statistic (F_ST_) was calculated in 1-kbp windows over each chromosome using the *windowed_weir_cockerham_fst* function in scikit-allel^97^.

### Examination of insecticide resistance associated genes

A bed file of genes commonly associated with target-site insecticide resistance SNPs was created. All annotated cytochrome P450s and esterases linked to metabolically mediated insecticide resistance were included. The bed file was then applied to the multi-sample VCF using *bcftools* (v1.17) view function. The snpEff software was then used to annotate the effect each SNP would have on the protein’s amino acid sequence^90^. A custom-built database was created for this, following the snpEff instructions at https://pcingola.github.io/SnpEff/snpeff/build_db_gff_gtf/ and using the instructions AnoDar_H01 GFF file which is available for download from NCBI https://www.ncbi.nlm.nih.gov/datasets/genome/GCF_943734745.1/). DELLY (v1.1.8) was used to investigate CNVs across the whole genome^103^. These were then filtered for quality and for those impacting genes within the expanded list of candidate genes used above.

### Ethics & inclusion

This project was determined and conducted in collaboration with local researchers and is locally relevant.

### Reporting summary

Further information on research design is available in the Nature Portfolio Reporting Summary linked to this article.

## Supplementary information

Supplementary Information

Description of Additional Supplementary File

Supplementary Data 1

Supplementary Data 2

Reporting Summary

## Data Availability

All raw data used in this work is publicly available from the NCBI BioProject database under accession number PRJEB66076. Sample IDs for the mosquito samples used in this study are provided in Supplementary Table 2.

## Code availability

All code used to analyse data is publicly available at https://github.com/sophiemoss/Anopheles_darlingi_genome_wide_analysis_Rondonia_Brazil.
