# Supplementary material for: Genome-wide analysis of genetic diversity in Anopheles darlingi from Rondônia State, Brazil
Source: Commun Biol. 2025 Dec 4;9:52. doi: 10.1038/s42003-025-09316-w (PMC12796178; doi:10.1038/s42003-025-09316-w)
Supplement: Supplementary file 1 — Supplementary Information [file 42003_2025_9316_MOESM1_ESM.pdf]

Supplementary Information

Principal Components Analyses

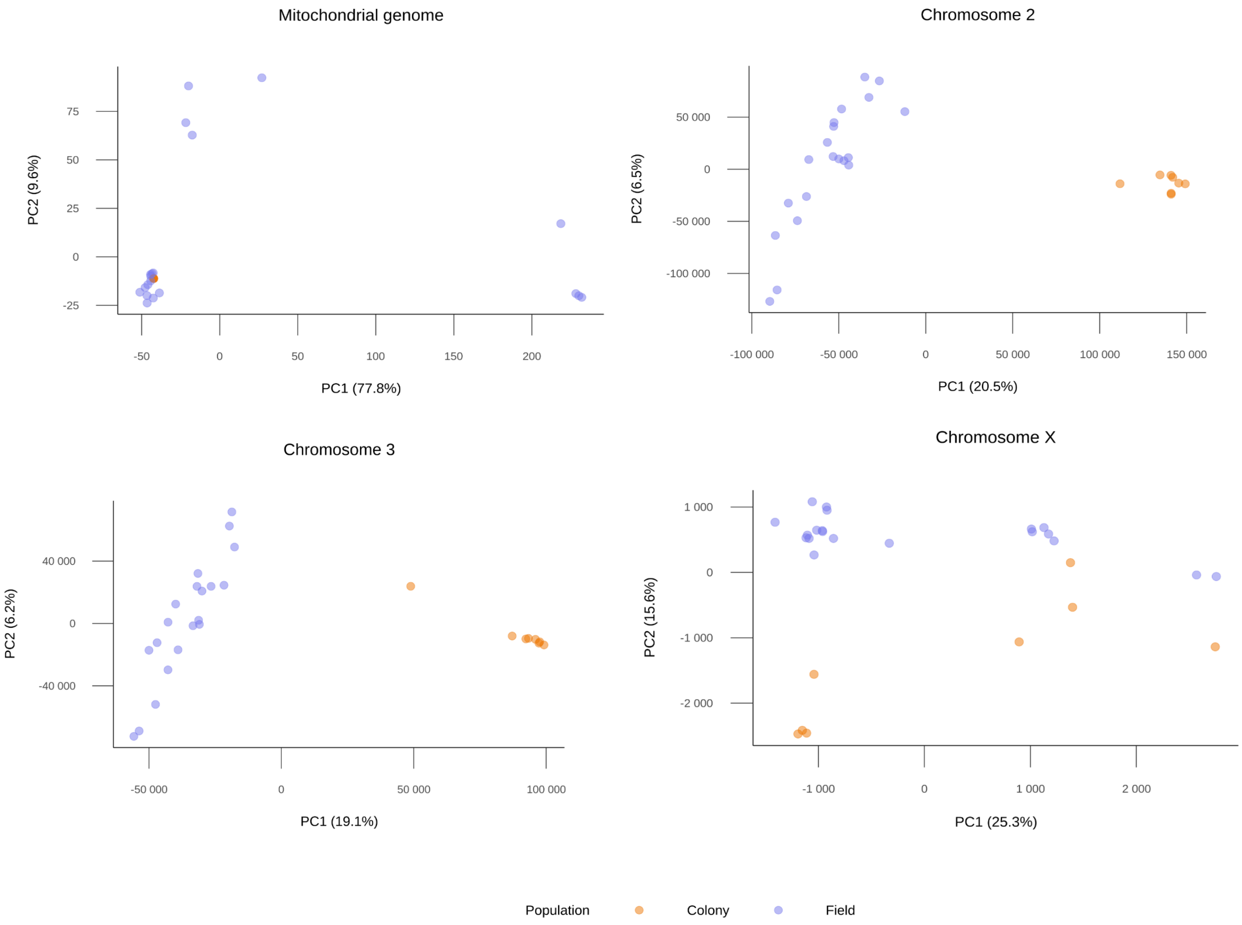

Supplementary Figure 1. Principal Components Analysis per chromosome for colony and wild-caught mosquitoes. *An. darlingi* colony (n = 8) and wild (n = 20) mosquitoes.

Maximum Likelihood Tree

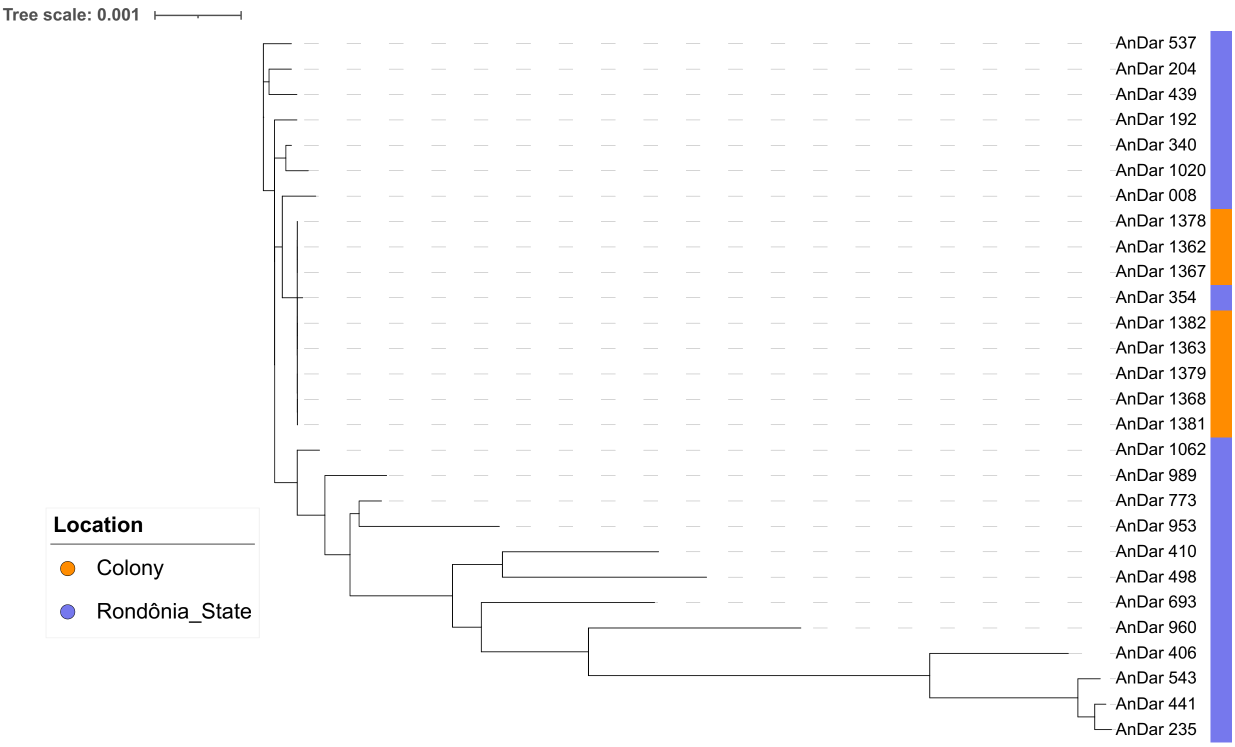

Supplementary Figure 2. Maximum likelihood tree of colony and wild-caught mosquito mitochondrial genomes. *An. darlingi* colony (n = 8) and wild (n = 20) mosquitoes.

Garud’s H12 Genome Wide Selection Scan

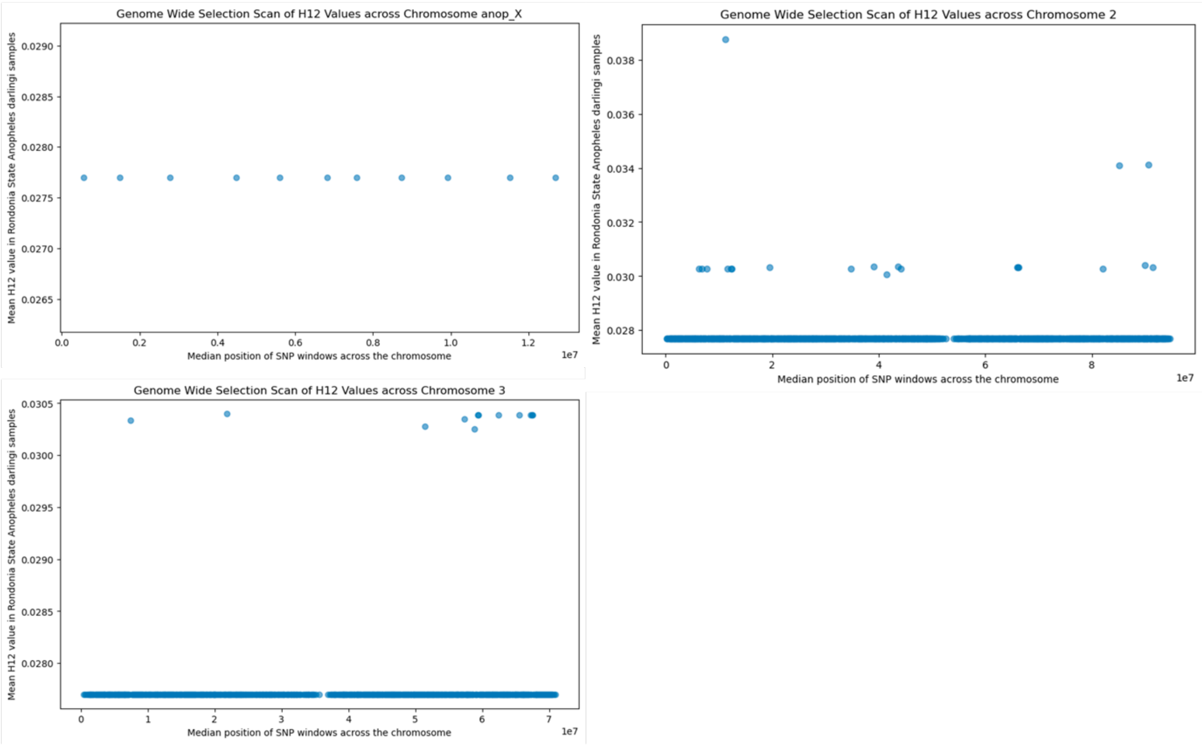

Supplementary Figure 3: Garud’s H12 statistic did not identify selective sweeps across any of the chromosomes in the wild-caught mosquitoes (n = 20).

Tajima's D

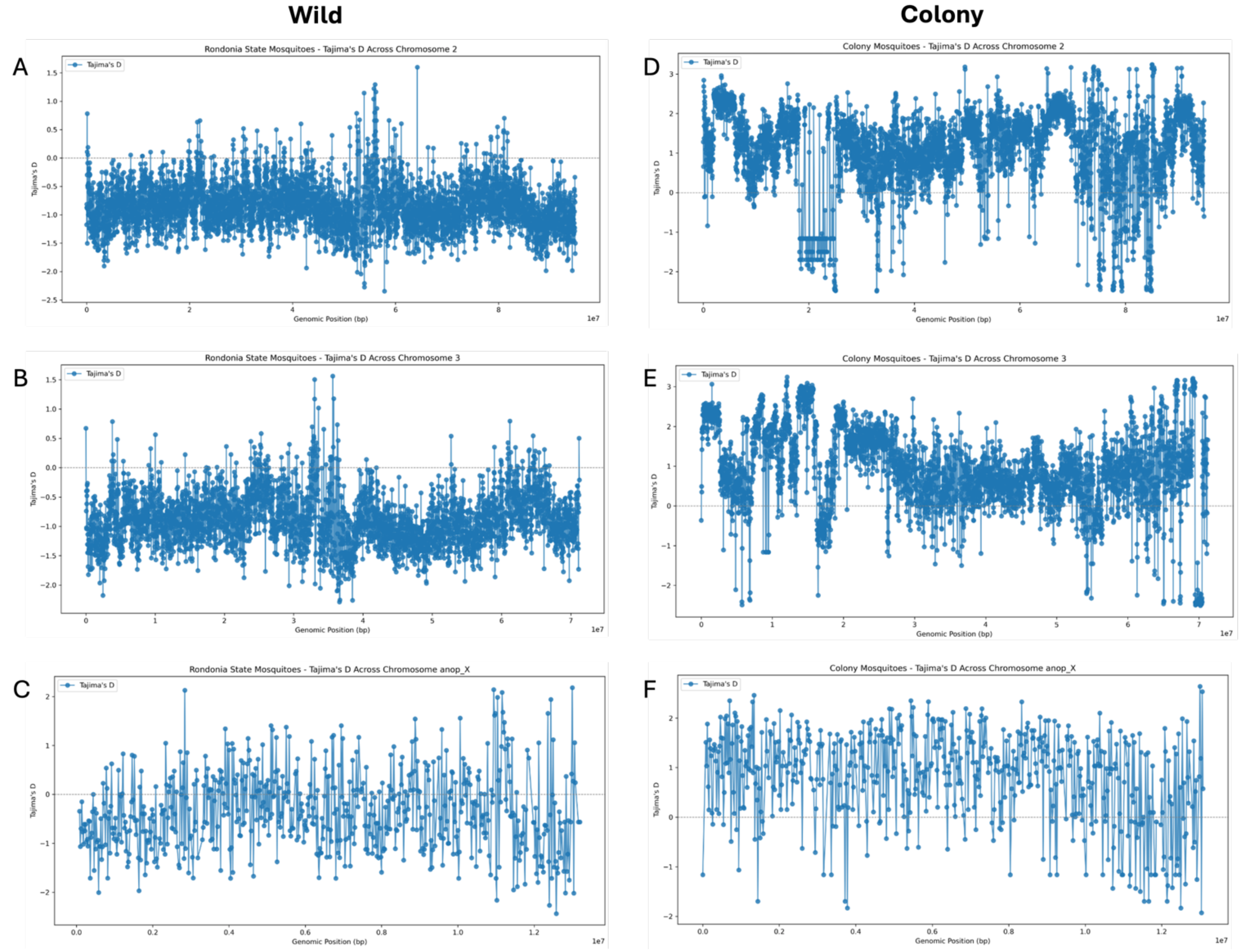

Supplementary Figure 4: Tajima's D scores for wild (n = 20) and colony (n = 8) mosquitoes calculated in 20 kb windows.

Supplementary Table 1: Four key insecticide resistance genes investigated and their locations in *An. darlingi*

Gene	Chr	Start	Stop	NCBI ID
ace1	NC_064874.1	15669736	15689194	LOC125950986
gste2	NC_064874.1	89825304	89827998	LOC125959620
vgsc	NC_064875.1	35305908	35339285	125955869
rdl	NC_064875.1	53378544	53446631	LOC125954302

Supplementary Table 2: Sample IDs used in this study.

Sample ID	Location, Rondônia State
AnDar_1362	Colony
AnDar_1363	Colony
AnDar_1367	Colony
AnDar_1368	Colony
AnDar_1378	Colony
AnDar_1379	Colony
AnDar_1381	Colony
AnDar_1382	Colony
AnDar_008	Wild
AnDar_1020	Wild
AnDar_1062	Wild
AnDar_192	Wild
AnDar_204	Wild
AnDar_235	Wild
AnDar_340	Wild
AnDar_354	Wild
AnDar_406	Wild
AnDar_410	Wild
AnDar_439	Wild
AnDar_441	Wild
AnDar_498	Wild
AnDar_537	Wild
AnDar_543	Wild
AnDar_693	Wild
AnDar_773	Wild
AnDar_953	Wild
AnDar_960	Wild
AnDar_989	Wild
