# Supplementary material for: Genome-wide analysis of genetic diversity in Anopheles darlingi from Rondônia State, Brazil
Source: Commun Biol. 2025 Dec 4;9:52. doi: 10.1038/s42003-025-09316-w (PMC12796178; doi:10.1038/s42003-025-09316-w)
Supplement: Supplementary file 2 — Description of Additional Supplementary File [file 42003_2025_9316_MOESM2_ESM.pdf]

Description of Additional Supplementary Files

File name: Supplementary Data 1

Description: Non-synonymous SNPs identified in the mosquito population.

File name: Supplementary Data 2

Description: Structural variants identified in the mosquito population.
