# Supplementary material for: Genome-wide analysis of genetic diversity in Anopheles darlingi from Rondônia State, Brazil
Source: Commun Biol. 2025 Dec 4;9:52. doi: 10.1038/s42003-025-09316-w (PMC12796178; doi:10.1038/s42003-025-09316-w)
Supplement: Supplementary file 5 — Reporting Summary [file 42003_2025_9316_MOESM5_ESM.pdf]

nature portfolio

Corresponding author(s): Sophie Moss
Last updated by author(s): Nov 13, 2025

Reporting Summary

Nature Portfolio wishes to improve the reproducibility of the work that we publish. This form provides structure for consistency and transparency in reporting. For further information on Nature Portfolio policies, see our Editorial Policies and the Editorial Policy Checklist.

Statistics

For all statistical analyses, confirm that the following items are present in the figure legend, table legend, main text, or Methods section.

n/a	Confirmed	
☐	☒	The exact sample size (*n*) for each experimental group/condition, given as a discrete number and unit of measurement
☐	☒	A statement on whether measurements were taken from distinct samples or whether the same sample was measured repeatedly
☐	☒	The statistical test(s) used AND whether they are one- or two-sided<br>*Only common tests should be described solely by name; describe more complex techniques in the Methods section.*
☐	☒	A description of all covariates tested
☐	☒	A description of any assumptions or corrections, such as tests of normality and adjustment for multiple comparisons
☐	☒	A full description of the statistical parameters including central tendency (e.g. means) or other basic estimates (e.g. regression coefficient) AND variation (e.g. standard deviation) or associated estimates of uncertainty (e.g. confidence intervals)
☐	☒	For null hypothesis testing, the test statistic (e.g. *F*, *t*, *r*) with confidence intervals, effect sizes, degrees of freedom and *P* value noted<br>*Give P values as exact values whenever suitable.*
☒	☐	For Bayesian analysis, information on the choice of priors and Markov chain Monte Carlo settings
☒	☐	For hierarchical and complex designs, identification of the appropriate level for tests and full reporting of outcomes
☒	☐	Estimates of effect sizes (e.g. Cohen's *d*, Pearson's *r*), indicating how they were calculated

Our web collection on statistics for biologists contains articles on many of the points above.

Software and code

Policy information about availability of computer code

Data collection

No software was used to collect the data.

Data analysis

Whole Genome Sequencing, and Bioinformatics Analysis
Twenty-eight An. darlingi isolates were sequenced using the Illumina MiSeq on 2 x 250bp paired end configuration. The raw paired fastq files were trimmed using trimmomatic software (version 0.39), and then aligned using bwa-mem software to the AnoDar_H01 (An. darlingi) reference genome using default parameters. Genome coverage from the mapped bam files was calculated using samtools, and variants called and validated using HaplotypeCaller and VailidateVariants from GATK software respectively (v4.1.4.1). Once VCFs had been generated for each sample, GATK was used to create a multi-sample VCF with GenomicsDBImport and GenotypeGVCF functions. The multi-sample VCF was filtered to include chromosomal variants using bcftools (v 1.17) and GATK's VariantFiltration, with parameters: QD > 5.0, QUAL > 30.0, SOR < 3.0, FS < 60.0, MQ > 40.0, MQRankSum > – 12.5, ReadPosRankSum > -8.0. Reads were subsequently filtered to retain those with DP > 5.0 and GQ > 20.0, and variants were filtered to retain those with < 20.0% missing genotypes or MAF > 0.01. The resulting VCF was phased with Beagle (version 5.2)72 and annotated using SnpEff (v5.1d).

Population Genetic Analysis
From the filtered multi-sample VCF, a pairwise-genetic distance matrix was generated using PLINK (v1.90b6.21 64-bit). This matrix was used for the generation of a maximum-likelihood tree using RAxML-NG (v 1.2.0), and principal component analysis was calculated using the R package ape (5.7-1). The ML tree generated was annotated and visualised in iTOL. Ancestry admixture analysis was conducted using ADMIXTURE software (version 1.3). The optimum K value (estimated number of ancestral populations) was calculated through cross-validation of 1-10 eigenvalue decay dimensions. In this instance, K = 2 was estimated, and ADMIXTURE software was used to analyse the shared ancestral populations (denoted K1 and K2) in these samples. The output was then visualised in R using ggplot2 package (v 3.4.2).

Genomic regions under selection were identified with the python package scikit-allel (v 1.3.6). Three complementary statistics were used: H12, iHS and XP-EHH. The Integrated Haplotype Statistic (iHS) was used to find directional selection within populations, and extended haplotype homozygosity (XP-EHH) between the two populations. Garud's H12 was calculated using phased biallelic SNPs in 1000 bp windows, using the moving_garud_h function. Two hundred iterations of H12 were calculated, followed by plotting of the mean value for each genomic window. iHS was computed using phased biallelic SNPs using the allel.ihs function79. Raw iHS scores were then standardized using the function allel.standardize_by_allele_count and p-values were plotted. XP-EHH was calculated using phased biallelic SNPs using the allel.xpehh function. The Tajima's D (TD) statistic was calculated using vcftools (v0.1.16) in 20 kb windows across chromosomes to identify balancing selection. Nucleotide diversity (π) was calculated in 100 bp windows across chromosomes using vcftools and plotted in R. Weir and Cockerham's F statistic (FST) was calculated in 1-kbp windows over each chromosome using the windowed_weir_cockerham_fst function in scikit-allel.

Examination of Insecticide Resistance Associated Genes
A bed file of genes commonly associated with target-site insecticide resistance SNPs was created. All annotated cytochrome P450's and esterase's linked to metabolically mediated insecticide resistance were included. The bed file was then applied to the multi-sample VCF using bcftools (v1.17) view function. The snpEff software was then used to annotate the effect each SNP would have on the protein's amino acid sequence44. A custom-built database was created for this, using the AnoDar_H01 GFF file. DELLY (v1.1.8) was used to investigate CNVs across the whole genome. These were then filtered for quality and for those impacting genes within the expanded list of candidate genes used above.

For manuscripts utilizing custom algorithms or software that are central to the research but not yet described in published literature, software must be made available to editors and reviewers. We strongly encourage code deposition in a community repository (e.g. GitHub). See the Nature Portfolio guidelines for submitting code & software for further information.

Data

Policy information about availability of data

All manuscripts must include a data availability statement. This statement should provide the following information, where applicable:
- Accession codes, unique identifiers, or web links for publicly available datasets
- A description of any restrictions on data availability
- For clinical datasets or third party data, please ensure that the statement adheres to our policy

All raw data used in this work is publicly available (NCBI: https://www.ncbi.nlm.nih.gov/bioproject/PRJEB66076/). All code is available at https://github.com/sophiemoss/Anopheles_darlingi_genome_wide_analysis_Rondonia_Brazil.

Research involving human participants, their data, or biological material

Policy information about studies with human participants or human data. See also policy information about sex, gender (identity/presentation), and sexual orientation and race, ethnicity and racism.

Reporting on sex and gender	Not applicable.
Reporting on race, ethnicity, or other socially relevant groupings	Not applicable.
Population characteristics	Not applicable.
Recruitment	Not applicable.
Ethics oversight	Not applicable.

Note that full information on the approval of the study protocol must also be provided in the manuscript.

Field-specific reporting

Please select the one below that is the best fit for your research. If you are not sure, read the appropriate sections before making your selection.

☐ Life sciences ☐ Behavioural & social sciences ☒ Ecological, evolutionary & environmental sciences

For a reference copy of the document with all sections, see nature.com/documents/nr-reporting-summary-flat.pdf

Ecological, evolutionary & environmental sciences study design

All studies must disclose on these points even when the disclosure is negative.

Study description	Genome-wide analysis of genetic diversity in Anopheles darlingi. In this study, we perform whole genome analysis of individual An. darlingi mosquitoes to explore genomic diversity, signatures of selection, and identify insecticide resistance markers. We analysed field-collected (n=20) and colony-maintained (n=8) mosquitoes from the State of Rondônia, Brazil.

Research sample	We analysed field-collected (n=20) and colony-maintained (n=8) mosquitoes from the State of Rondônia, Brazil. This is one of the largest studies of this mosquito species by whole genome sequencing to date.
Sampling strategy	Two populations of An. darlingi from the State of Rondônia were used in this study, one wild-caught population from Candeias do Jamari, right margin of Madeira River, (n = 20), and a cohort of colony isolates from Porto Velho (n = 8). The wild-caught mosquitoes were collected during vector density studies in malaria-endemic regions in Rondônia in 2018-19. The colony mosquitoes have been maintained since in 2018, originally collected in Porto Velho, left margin of Madeira River.
Data collection	This study generates WGS data and analyses this for previously collected mosquitoes. Two populations of An. darlingi from the State of Rondônia were used in this study, one wild-caught population from Candeias do Jamari, right margin of Madeira River, (n = 20), and a cohort of colony isolates from Porto Velho (n = 8). The wild-caught mosquitoes were collected during vector density studies in malaria-endemic regions in Rondônia in 2018-19. The colony mosquitoes have been maintained since in 2018, originally collected in Porto Velho, left margin of Madeira River.
Timing and spatial scale	The wild-caught mosquitoes were collected during vector density studies in malaria-endemic regions in Rondônia in 2018-19. The colony mosquitoes have been maintained since in 2018, originally collected in Porto Velho, left margin of Madeira River. Entomological surveys were performed as 8 consecutive collections at the beginning of the rainy season (Oct and Nov 2018) and another eight collections were performed at the beginning of the dry season (May and June 2019) (https://pmc.ncbi.nlm.nih.gov/articles/PMC8323833/).
Data exclusions	Data was not excluded from the analyses. A subset of collected mosquitoes were selected for WGS and analysis due to the cost constraints of sequencing.
Reproducibility	Mosquitoes were collected during entomological surveillance surveys using standard collecting methods which are reproducible. All genomic data has been made publicly available, along with code for analyses.
Randomization	Not relevant to this study, as a subset of collected mosquitoes were used for WGS and analysis.
Blinding	Not relevant to this study, as no blinding was required; a subset of mosquitoes were used for WGS and analysis.

Did the study involve field work? ☐ Yes ☒ No

Reporting for specific materials, systems and methods

We require information from authors about some types of materials, experimental systems and methods used in many studies. Here, indicate whether each material, system or method listed is relevant to your study. If you are not sure if a list item applies to your research, read the appropriate section before selecting a response.

Materials & experimental systems

n/a	Involved in the study
☒	☐ Antibodies
☒	☐ Eukaryotic cell lines
☒	☐ Palaeontology and archaeology
☒	☐ Animals and other organisms
☒	☐ Clinical data
☒	☐ Dual use research of concern
☒	☐ Plants

Methods

n/a	Involved in the study
☒	☐ ChIP-seq
☒	☐ Flow cytometry
☒	☐ MRI-based neuroimaging

Plants

Seed stocks	Not applicable.
Novel plant genotypes	Not applicable.
Authentication	Not applicable.
